# An investigation of the longitudinal trajectory patterns of health-related quality of life among Australians with disabilities: explaining disability types and properties

**DOI:** 10.1007/s11136-024-03683-3

**Published:** 2024-06-10

**Authors:** Rubayyat Hashmi, Byron W. Keating, Mohammad Afshar Ali, Syed Afroz Keramat

**Affiliations:** 1 The Australian Centre for Housing Research, The University of Adelaide, Adelaide, SA 5005 Australia; 2 QUT Business School, Queensland University of Technology, Brisbane, QLD 4001 Australia; 3 Centre for Health Services Research, Faculty of Medicine, University of Queensland, Brisbane, QLD Australia; 4 The ALIVE National Centre for Mental Health Research Translation, The University of Melbourne, Melbourne, Australia; 5 Sydney Pharmacy School, The University of Sydney, Sydney, Australia

**Keywords:** Disability, HRQoL, GBTM, Australia

## Abstract

**Background:**

Research on health-related quality of life (HRQoL) trajectory patterns for people with disabilities (PwD) is scant. Understanding the HRQoL trajectory patterns for PwDs and investigating their relationship with disability types and socioeconomic factors can have important implications for Australia’s welfare policy.

**Methods:**

We analysed data from waves 11 to 21 of the Household, Income and Labour Dynamics in Australia (HILDA) survey of respondents aged 15 + years of the PwDs. The analytic sample consists of 3724 self-reported disabled individuals and 34,539 observations in total. The SF-6D utility score is our HRQoL measure. Group-based trajectory modelling was utilised to identify trajectory groups, and multinomial logistic regression was employed to determine the baseline factors associated with trajectory group membership.

**Results:**

The study identified four distinct types of HRQoL trajectories (high, moderate improving, moderate deteriorating and low HRQoL trajectories). Psychosocial disability types followed by physical disability types had a high Relative Risk Ratio (RRR) in the low group compared with high trajectory group membership of PwDs (psychosocial: 6.090, physical: 3.524). Similar, results followed for the moderate improving group albeit with lower RRR (psychosocial: 2.868, Physical: 1.820). In the moderate deteriorating group, the disability types were not significant as this group has a similar profile to high group at the baseline. Compared with males, females had a higher RRR in low and moderate versus high improving HRQoL trajectories (low: 1.532, moderate improving: 1.237). Comparing the richest class to the poorest class, socioeconomic factors (income and education) predicted significantly lower exposure for the richer class to the low and medium HRQoL trajectories groups (RRR < 1).

**Conclusion:**

Different forms of disability, demographic and socioeconomic factors have distinct effects on the HRQoL trajectories of disabled individuals. Healthcare and economic resource efficiency might be improved with targeted government policy interventions based on disability trajectories.

**Supplementary Information:**

The online version contains supplementary material available at 10.1007/s11136-024-03683-3.

## Introduction

Albrecht and Devlieger describe the ‘disability paradox’, which questions why so many people with disabilities (PwD) report a high quality of life even though the undesirable nature of a disability condition would lead one to anticipate that PwD would report a low quality of life [1]. Koch questioned the existence of this paradox and suggested that social support, financial resources, personal perspective or coping mechanisms could explain this phenomenon [2]. Numerous works have since attempted to explain the ‘disability paradox’ [3–6] using cross-sectional methods to study disability at a single point in time. The present research adds to the literature through a longitudinal evaluation of how preference-based health-related quality of life (HRQoL) evolves over time.

Prior work has explored various dimensions of well-being following the onset of disability, suggesting that disability onset can negatively impact subjective well-being and mental health, with some experiencing long-term decline [7, 8]. Studies have also shown that young adults with childhood-onset often experience functional limitations and lower quality of life [9], with the impact of disability on life satisfaction shown to vary by age overtime. In particular, individuals experiencing disability at younger or older ages tend to report steeper declines compared to those disabled in mid-life [10]. The present research adds to this literature by exploring HRQoL trajectories among PwD. Specifically, we will seek to understand the ‘disability paradox’ through HRQoL trajectories using Australian population data.

It is estimated that over 4 million people or approximately 18% of the population, in Australia live with a disability [11]. Australian Institute of Health and Welfare (AIHW) data shows that, a significant proportion of PwD need some form of care: 30% require assistance in managing their healthcare needs, 27% necessitate aids in maintaining their property and 23% rely on support for various home activities [11]. To achieve the best outcomes for PwD, the Australian government has outlined a 10-year disability strategy targeting healthcare and improved support services via the National Disability Insurance Scheme (NDIS) [12]. Additionally, the Disability Support Pension (DSP) which provides financial help for PwD with permanent conditions that prevent working. The NDIS complements this financial support with a market-based scheme that fosters individual-focused support, to enhance independence and socioeconomic engagement. However, recent evaluations of the NDIS have raised questions about its effectiveness in raising the quality of life for PwD [13–15].

The objective of this paper is two-fold. First, we seek to identify the number of distinct HRQoL group trajectories for PwD to verify whether a ‘disability paradox’ exists. Second, we will investigate the characteristics of individuals within the different group trajectories (e.g., clinical factors such as disability types and disability properties) to identify targeted policy recommendations. This is critical at a time where there is political pressure to effectively allocate scarce resources for maximum benefit.

## Methods

### Data source and sample selection

The Household, Income and Labour Dynamics in Australia (HILDA) is a nationally representative broad socioeconomic longitudinal survey for Australian residents. Other comparable surveys in the world like HILDA include the US Panel Study of Income Dynamics (PSID), the UK Household Longitudinal Study (UKHLS), and the German Socio-Economic Panel (SOEP). Readers interested in learning more about the HILDA survey might check the HILDA user manual or Nicol Watson's review of the HILDA survey [16, 17].

**Fig. 1:**
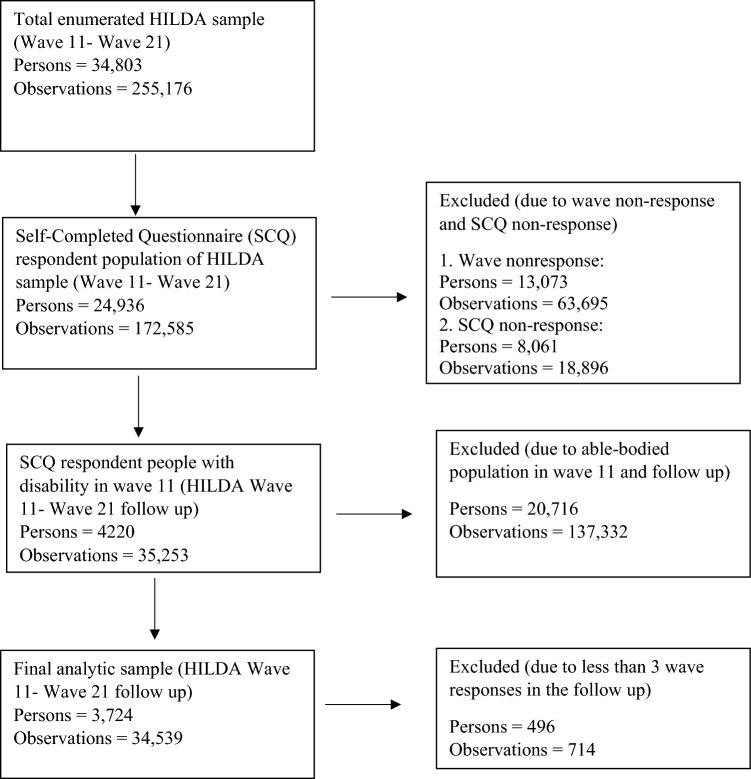
Participants flow into the analytic sample

The HILDA survey incorporated a top-up sample of 2000 households in 2011 to alleviate the impact of sample attrition and uphold population representativeness amidst shifting demographics in Australia. Therefore, wave 11 (2011) was selected as the baseline year for our analysis. This study analyzed individuals who self-reported a disability in wave 11 of HILDA survey data and followed up on those individuals up to wave 21 (Individuals who responded fewer than three times were excluded, as at least three data points are needed to understand a trajectory). This resulted in 3724 individuals and 34,539 observations for our study (i.e., 3724 individuals reported having a disability in 2011 and took part in the subsequent surveys at least three times. Individuals who did not report a disability in 2011 but later developed one, or those who had a disability previously but did not report it in 2011, are excluded from our analytical sample). A sequential imputation strategy addresses missing data in individual responses (item-wise). We prioritize forward imputation, where the missing value is estimated based on the respondent’s most recent preceding response. In cases where forward imputation is not applicable (i.e., the missing value is at the beginning of the sequence), we employ backward imputation, utilizing the respondent’s closest following response. The participant flow into the analytic sample is shown in Fig. 1.

### Outcome measure

The existing literature employs a variety of Health-Related Quality of Life (HRQoL) instruments to assess health and well-being for both individuals with and without disabilities (e.g., EQ-5D, ASCOT, AQoL, SF-6D etc.)[18, 19]. Since the HILDA survey provides only one measure of preferences-based HRQoL: SF-6D, we used it as our outcome measure. The SF-6D utility score, a widely used preference-based HRQoL measure was developed by Brazier and colleagues [20]. Utilizing the six domains (six physical functioning items, four role limitations items, five social functioning items, six pain items, five mental health items, and five vitality items) of the SF-36 instrument [21], the SF-6D is capable of predicting utility scores for 18,000 health states (6 × 4 × 5 × 6 × 5 × 5). In our study, the SF-6D is the primary outcome variable. Our study sample includes 641 distinct health states, with scoring ranges from 0.301 to 1.

### Disability measure

The HILDA survey collects participants’ disability status through a face-to-face interview. Participants who reported disability conditions at wave 11 were selected for this study and followed longitudinally. Using the guidelines provided by earlier studies [22–24], this study classified the 17 different types of disability into four major categories: (I) Sensory disability, (II) Physical disability, (III) Psychosocial disability, and (IV) Other disability or long term conditions. The study also considers whether the individuals have single or multiple disabilities and whether disability status is work-limiting or not. Details of the disability types are provided in Table A1 of the supplementary material (Appendix A).

**Table 1:** Sample demographics characteristics

Variables	wave 11	wave 12	wave 13	wave 14	wave 15	wave 16	wave 17	wave 18	wave 19	wave 20	Wave 21	Total
*Participants*												
n	3724	3534	3507	3395	3247	3213	3060	2929	2828	2619	2483	34,539
%	10.78	10.23	10.15	9.83	9.40	9.30	8.86	8.48	8.19	7.58	7.19	100
*Gender*												
Male (n)	1659	1577	1563	1509	1443	1418	1341	1280	1231	1132	1076	15,229
(%)	44.55	44.62	44.57	44.45	44.44	44.13	43.82	43.70	43.53	43.22	43.33	44.09
Female (n)	2065	1957	1944	1886	1804	1795	1719	1649	1597	1487	1407	19,310
(%)	55.45	55.38	55.43	55.55	55.56	55.87	56.18	56.30	56.47	56.78	56.67	55.91
*Age*												
15–24 (n)	301	239	217	186	141	122	86	58	41	18	0	1409
(%)	8.08	6.76	6.19	5.48	4.34	3.80	2.81	1.98	1.45	0.69	0.00	4.08
25–54 (n)	1378	1230	1177	1105	1026	1048	970	906	842	789	740	11,211
(%)	37.00	34.80	33.56	32.55	31.60	32.62	31.70	30.93	29.77	30.13	29.80	32.46
55–64 (n)	844	807	779	749	712	696	673	648	617	563	529	7617
(%)	22.66	22.84	22.21	22.06	21.93	21.66	21.99	22.12	21.82	21.50	21.30	22.05
65 + (n)	1201	1258	1334	1355	1368	1347	1331	1317	1328	1249	1214	14,302
(%)	32.25	35.60	38.04	39.91	42.13	41.92	43.50	44.96	46.96	47.69	48.89	41.41

### Other covariates

In addition to the above-mentioned clinical factors, the study also includes demographic covariates (age and gender) and socioeconomic covariates (equivalized household income quintile, education and current labour force status) to comprehend the relationship between the various trajectory groups identified from our HRQoL measure.

### Statistical analyses

To begin, we described the study participants’ demographic characteristics. We used group-based trajectory modeling (GBTM) to identify subgroups of PwD who share similar quality-of-life trajectories based on their SF-6D health state utility scores at each point [25–27]. Following group identification, we examined the distribution of disability types over time and estimated the transition probabilities of disability conditions for each group (STATA command “xttrans” is used to estimate transition probabilities). Finally, we assessed demographic, clinical and socioeconomic factors’ association with quality-of-life trajectory groups through a multinomial logistic regression technique.

#### Group-based trajectory modeling

Group-based trajectory modeling by Nagin has extensive application in several fields such as psychology, criminology and clinical/medical research [26, 28]. The approach uses finite mixture of probability distributions to discover cluster of individual trajectories with similar patterns in phenomena generally connected to behavioural or health outcome [29–31]. Individuals within identified groups can then be profiled based on their characteristics [29]. Maximum likelihood is used to estimate GBTM parameters [29–31]. Details of the GBTM methodology is provided in the supplementary material (Appendix B).

#### Estimation strategy and model selection criteria

The analyses were performed with the STATA/MP version 18 statistical software together with the Traj plugin for STATA [29]. The key model selection decision in GBTM is to determine the number of groups or latent classes of group trajectories. Bayesian Information Criteria (BIC), Akaike Information Criteria (AIC) and entropy are the most frequently employed test statistic for evaluating model fit [28]. We followed Nagin’s recommendation of using Bayes Factor in assessing initial model fit [31]. Changes in BIC between models can act as an approximation of log Bayes factor, which can be used to determine the number of groups [32, 33]. The test statistic indicates the degree of support for the alternative model over the simplified model [31]. However, Nagin also mentioned that implementing a formal statistical criterion rigidly may result in inferior selection [28]. Thus, he proposed additional objective standards for the model selection: (1) average posterior probabilities of group membership exceeds a minimum threshold of 0.7 and (2) Odds of correct classification of group membership exceeds a minimum threshold of 5. Other studies also used (1) a minimum entropy threshold of 0.8 and (2) a minimum sample size of 5% for each identified groups [34, 35]. Estimation procedure details are provided in the supplementary material (Appendix B).

## Results

Table 1 summarizes the demographic characteristics of the study participants across waves 11–21 of the HILDA survey. After ten waves, approximately two-thirds of the study participants were retained in our study sample. The sex ratio varies between 0.76 to 0.80 males/female over this period. 58.59% of our sample observations are from working-age population, and the rest are elderly.

### Identification of HRQoL trajectories and groups for people with disabilities (PwD)

Table 2 tabulates the BIC, Bayes Factor (B_10_), entropy, group size, average posterior probability (APP) and Odds of Correct Classification (OCC) for model fits to the HRQoL data of PwD in HILDA survey. We know that, if the Bayes factor is greater than the value of 150 (or 2log_e_B_10_ > 10), the evidence against the null model is very strong [25]. Table 2 shows that the number of classes with Bayes factor threshold value greater than 150 is seven classes. However, from seven class trajectories, the entropy is also lower than 0.8, and the smallest group size fell below 5%, violating our threshold criteria. For this reason, we determined that, there are six trajectory classes of HRQoL for people with disabilities.

**Table 2:** Model selection criteria statistics for the number of classes (trajectories)

						Lowest		
Number of classes	Null model	BIC	B_10_	2log_e_B_10_	Entropy	Group size	APP	OCC
1		21,309.21						
2	1	29,731.19	8421.98	18.08	0.91	44.51	0.97	31.09
3	2	32,118.05	2386.86	15.56	0.87	29.17	0.92	17.71
4	3	33,133.93	1015.88	13.85	0.85	11.30	0.90	18.55
5	4	33,576.53	442.60	12.19	0.82	7.16	0.86	16.02
6	5	33,874.97	298.44	11.40	0.80	7.84	0.79	17.88
7	6	34,068.82	193.85	10.53	**0.77**	**4.45**	0.80	11.70
8	7	34,192.10	**123.28**	**9.63**	**0.77**	**3.29**	0.77	12.55

**Fig. 2:**
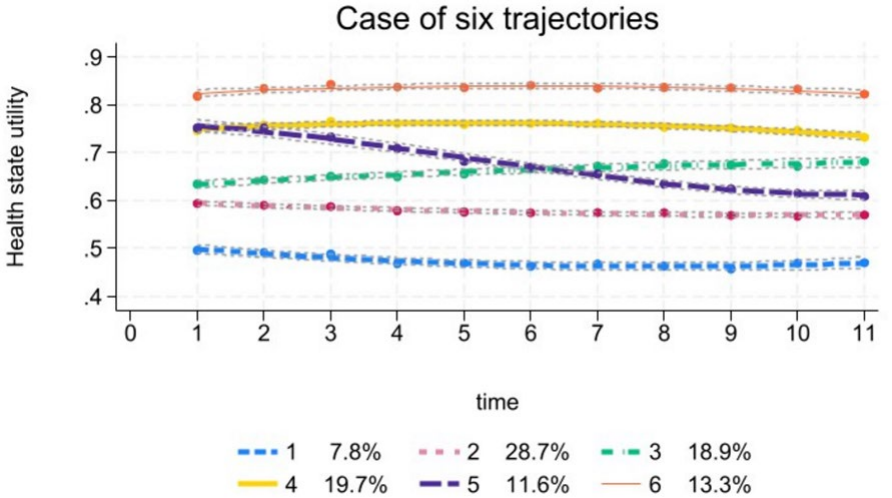
Six-trajectory types of health state utilities among disabled population of Australia

Figure 2 depicts the six HRQoL trajectories for PwD. From the graph, we can see that Trajectory 1 and 2 both exhibit a low HRQoL trajectory throughout the study. We classified the individuals exhibiting these two trajectories as the low HRQoL trajectory group. Similarly, individuals exhibiting Trajectories 4 and 6 exhibit a high HRQoL trajectory throughout the study period and we classified this group as the High HRQoL trajectory group. Trajectory 3 started with lower than the High trajectory group but gradually improved over the study period. We classified individuals following this trajectory as the Moderate-Improving HRQoL trajectory group. Finally, Trajectory 5 almost started like Trajectory 4, but gradually deteriorated and had lower HRQoL than Trajectory 3 (Moderate-Improving group) after six years. We defined individuals following this trajectory group as Moderate-Deteriorating HRQoL trajectory group. Hence, the four distinct groups form these six trajectories are: (1) Group 1: High HRQoL trajectory group (33% of the population) (2) Group 2: Moderate-Improving HRQoL trajectory group (18.9% of the population) (3) Group 3: Moderate-Deteriorating HRQoL trajectory group (11.6% of the population) and, (4) Group 4: Low HRQoL trajectory group (36.5% of the population).

### Descriptive analyses

**Fig. 3:**
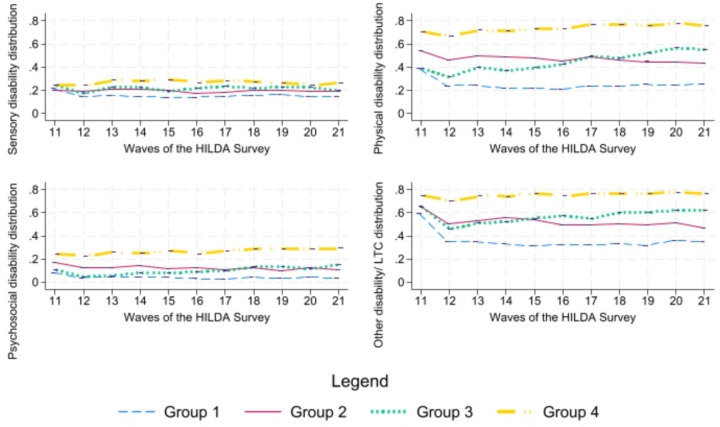
Distribution of disability-types overtime by HRQoL trajectory groups (Group 1: High, Group 2: Moderate—Improving, Group 3: Moderate—Deteriorating, and Group 4: Low)

Figure 3 illustrates the distribution of four types of disabilities (sensory, physical, psychosocial, and other) among the four HRQoL trajectory groups (Group 1: High, Group 2: Moderate-Improving, Group 3: Moderate-Deteriorating, and Group 4: Low). Group 4 has a high distribution trend for all four major disability categories (sensory: 24–29%, physical 67–78%, psychosocial: 23–29% and other: 70–78%). On the contrary, Group 1 has a low distribution trend for all four major disability categories (sensory: 14–22%, Physical: 21–39%, psychosocial: 3–8%, and other: 31–60%). Group 2 (sensory: 17–21%, physical 43–55%, psychosocial: 10–17% and other: 47–66%) usually has a higher distribution than Group 3 (sensory: 17–24%, physical 31–57%, psychosocial: 5–15% and other: 45–66%) at the beginning in physical, psychosocial, and other disabilities and the trend gradually dropped compared to Group 3. From wave 15 and onwards, Group 3 usually has a higher disability distribution than Group 2 and gradually increases. This makes sense, as, Group 3’s HRQoL dropped over time. Upon careful examination of the graphs, it becomes intriguing to observe that disability prevalence closely reflects the trajectory groups. Trajectories characterized by high disability prevalence are negatively related to low HRQoL scores.

The implication of distribution trends suggests that the absence, presence, or accumulation of disability conditions may have significantly affect an individual’s HRQoL. Table 3 provides the transition probabilities of disability status (no condition, single disability condition and multiple disability condition). Transition probabilities are the likelihood of transitioning from one state (e.g. single disability condition) to another (e.g., multiple disability condition). Transition counts between observations are used to estimate transition probabilities. Overall, there is a 65% chance that a person with no condition would have no more condition in the next period. Similarly, there is a 45% chance that an individual with a single disability condition would stay at the single disability condition in the next period and a 73% chance that a person with multiple disability conditions would stay at multiple disability conditions. A person with a single disability has a 30% probability of transitioning to multiple disabilities and a 25% probability of transitioning to a disability condition in the following period. A person with multiple disabilities has about a 20% probability of transitioning back to a single disability and approximately 7.5% of transitioning to no condition in the subsequent period. For the trajectory groups, Group 1 has a higher rate of transitioning to a better condition than Group 2, Group 2 has a higher transition rate to a better condition than Group 3, and so on.

**Table 3:** Estimated transition probabilities by disability status

		Disability status		
	Disability status	No condition	Single disability	Multiple disability
All	No condition	65.25 (4797)	23.57 (1733)	11.18 (822)
	Single disability	25 (2508)	45.52 (4566)	29.47 (2956)
	Multiple disability	7.56 (1016)	18.99 (2551)	73.45 (9866)
Group 1	No condition	72.86 (3281)	20.87 (940)	6.26 (282)
	Single disability	34.32 (1417)	48.63 (2008)	17.05 (704)
	Multiple disability	18.57 (383)	29.87 (616)	51.55 (1063)
Group 2	No condition	59.08 (774)	27.33 (358)	13.59 (178)
	Single disability	24.25 (495)	45.71 (933)	30.03 (613)
	Multiple disability	10.63 (256)	23.16 (558)	66.21 (1595)
Group 3	No condition	53.69 (436)	28.82 (234)	17.49 (142)
	Single disability	24.14 (315)	42.45 (554)	33.41 (436)
	Multiple disability	10.29 (146)	24.24 (344)	65.47 (929)
Group 4	No condition	42.09 (306)	27.65 (201)	30.26 (220)
	Single disability	11 (281)	41.92 (1071)	47.08 (1203)
	Multiple disability	3.06 (231)	13.69 (1033)	83.24 (6279)

Frequencies are in the brackets

### Regression results

Table 4 examines the associations between the baseline (wave 11) demographic, clinical and socioeconomic factors and the trajectory groups. The Relative Risk Ratio (RRR) and the multinomial logistic regression statistics are utilized to profile individual characteristics. The RRR represents the exponentiated coefficient in a multinomial logistic regression model. It reflects the change in the relative risk of the outcome in the comparison group compared to the referent group. An RRR > 1 indicates the risk of outcome falling in the comparison group increases compared to the referent group, and vice versa. Group 1: High HRQoL trajectory group is the reference group to which other groups are compared. The significant factors of Group 2: Moderate-Improving HRQoL trajectory group vs the reference groups are as follows (RRR are in brackets): aged 25–44 years (2.054), 45–54 years (1.666), 65 + years (1.612), female (1.237), physical disability (1.820), psychosocial disability(2.868), other disability (1.572), work-limiting disability (2.005), income: middle (0.622), rich (0.635), richest (0.615), and not in the labour force (1.568). Significant factors for Group 3: Moderate-Deteriorating HRQoL trajectory group vs reference group are as follows: aged 65 + years (2.169), income: middle (0.666) and not in the labour force (1.402). This result is interesting because only three factors significantly differ from the High HRQoL reference group. Hence, it is reasonable to assume that Group 3 primarily comprises older individuals with disabilities who are presently not part of the workforce and are experiencing deteriorating health.

**Table 4:** Baseline (Wave 11) factors associated with trajectory group membership in the multinomial logistic regression

	Group 1: High (Ref)	Group 2: Moderate-Improving			Group 3: Moderate-Deteriorating			Group 4: Low		
	Variables	RRR		95% CI	RRR		95% CI	RRR		95% CI
Demographic	*Age (Ref: 15–24 years)*									
	25–44 years	2.05	***	(1.40–3.02)	1.09		(0.72–1.65)	3.82	***	(2.53–5.76)
	45–54 years	1.67	*	(1.11–2.51)	0.93		(0.59–1.46)	3.12	***	(2.04–4.79)
	65 + years	1.61	*	(1.05–2.46)	2.17	***	(1.41–3.33)	3.96	***	(2.58–6.08)
	*Gender (Ref: Male)*									
	Female	1.24	*	(1.01–1.51)	1.17		(0.93–1.48)	1.53	***	(1.27–1.85)
Clinical	*Disability types*									
	Sensory (Ref: No)									
	Yes	1.13		(0.73–1.74)	0.85		(0.50–1.42)	1.47	*	(1.00–2.18)
	*Physical (Ref: No)*									
	Yes	1.82	**	(1.17–2.83)	0.83		(0.49–1.43)	3.52	***	(2.33–5.32)
	*Psychosocial (Ref: No)*									
	Yes	2.87	***	(1.78–4.63)	1.39		(0.76–2.54)	6.09	***	(3.88–9.55)
	Other (ref: No)									
	Yes	1.57	*	(1.01–2.45)	0.95		(0.55–1.62)	2.44	***	(1.61–3.70)
	*Disability status (Ref: Single disability)*									
	Multiple disability	1.03		(0.60–1.75)	1.41		(0.75–2.67)	0.83		(0.51–1.36)
	*Work limiting disability (Ref: No)*									
	Yes	2.01	***	(1.61–2.50)	1.71		(0.92–1.50)	4.15	***	(3.29–5.23)
Socioeconomic	*Income quintile (Ref: Poorest)*									
	Poor	0.87		(0.63–1.20)	0.83		(0.57–1.21)	0.76		(0.57–1.02)
	Middle	0.62	**	(0.46–0.85)	0.67	*	(0.47–0.95)	0.49	***	(0.37–0.64)
	Rich	0.64	**	(0.46–0.88)	0.86		(0.60–1.23)	0.47	***	(0.35–0.64)
	Richest	0.62	***	(0.44–0.86)	0.74		(0.50–1.09)	0.36	***	(0.26–0.51)
	*Education (Ref: Year 12 or below)*									
	Certificate/diploma	1.01		(0.80–1.27)	1.04		(0.80–1.37)	0.89		(0.72–1.10)
	Bachelor or higher	0.97		(0.73–1.27)	0.75		(0.53–1.06)	0.63	***	(0.48–0.83)
	*Labour force status (Ref: Employed)*									
	Unemployed	1.50		(0.81–2.75)	1.69		(0.84–3.40)	2.43	**	(1.34–4.39)
	Not in the labour force	1.57	***	(1.22–2.01)	1.40	*	(1.04–1.90)	2.14	***	(1.68–2.72)
	Constant	0.10	***	(0.06–0.19)	0.24	***	(0.12–0.47)	0.02	***	(0.01–0.04)

*RRR* Relative Risk Ratio, *CI* Confidence Interval, * < 0.05, ** < 0.01, *** < 0.001

On the contrary, except for multiple disability status and certificate/diploma education, all factors are highly significant for Group 4: Low HRQoL group versus the reference group (multiple disability status is not significant in any of the groups). The significant factors and their RRR for Low HRQoL groups are as follows: age: 25–44 years (3.817), 45–54 years (3.121), 65 + years (3.959), female (1.532), sensory disability (1.474), physical disability (3.524), psychosocial disability (6.090), other disability (2.439), work-limiting disability (4.146), income: middle (0.487), income: rich (0.472), income: richest (0.363), bachelor or higher education (0.631), unemployed (2.428), and not in the labour force (2.140). In general, from the regression analysis, we can see that Group 4: Low HRQoL has higher RRR in demographic and clinical factors (risk of falling in Group 4 is higher than the Group 1) and lower RRR in socioeconomic factors for better SES (risk of falling in group 4 is lower than the group 1 for higher SES). Further, females have a higher RRR, indicating they are at risk of failing in Group 2 versus Group 1 or Group 4 vs Group 1, indicating vulnerability of disabled females as well as HRQoL improving capacity of females. Among clinical factors, psychosocial disability has the highest RRR for Group 4, followed by work-limiting disability and physical disability, implying having these factors greatly increases the risk of falling in Group 4. Furthermore, the RRRs in Group 4 (Low) exhibit a greater magnitude than those in Group 2 (Moderate-Improving), suggesting that all these factors pose a higher risk of being classified into Group 4 (Low) than Group 2 (Moderate-Improving). Lastly, as people’s health deteriorates with age, elderly people have a greater chance of falling into Group 3 (Moderate-Deteriorating) or Group 4 (Low), as they have a high RRR of falling into these groups. In summary, various types of disability, as well as socioeconomic and demographic status, have distinct effects on the HRQoL of people with disabilities.

## Discussion

Disability has a negative influence on the perceived physical and mental health of an individual. However, various types of disability have a differential impact on the trajectories of HRQoL over time. We have used a novel technique (GBTM) to show the HRQoL patterns among Australian adults living with disabilities. We utilized a generic preference-based utility score (SF-6D) to show the trajectory of HRQoL over time. We revealed four HRQoL trajectory groups using several model fit statistics (e.g., BIC, B10, 2LogeB10, entropy, group size, APP, and OCC). Our results showed that Group 1 maintains the highest HRQoL, followed by Group 2: Moderate-Improving HRQoL), Group 3 (Moderate-Deteriorating HRQoL), and Group 4 (Lowest HRQoL). Our findings also demonstrated that approximately 36.5% (Group 4) of the study population had a utility score of less than 0.6 over the study period. We also revealed that, during the study period, the trajectory Group 4 (Lowest HRQoL) has the highest proportion of people living with sensory (∼ 25 to 30%), physical (∼ 70 to 80%), psychosocial (∼ 25 to 30%), and other types of disabilities (∼ 70 to 80%). In addition, we examine the associations of four types of disabilities with poorer HRQoL groups. Furthermore, we investigated the relationship between disability types and trajectories of HRQoL at the baseline. The objective here is to establish a profile of individual characteristics at the baseline to facilitate the development of policy interventions tailored to specific targets.

We found evidence that if an individual acquires physical or psychosocial disabilities, their relative risk of being in the Moderate-Improving (Group 2), Moderate-Deteriorating (Group 3), and Lowest HRQoL (Group 4) increases incrementally. According to our findings, the relative risk ratio of the lowest HRQoL is significantly higher among adults with all types of disabilities at baseline compared to High HRQoL. We also found that the relative risk ratio of Moderate-Improving HRQoL Group is significantly higher among adults with physical, psychological, and other disabilities at baseline compared to High HRQoL. Interestingly, we found that the relative risk ratio of the Moderate-Deteriorating HRQoL is not significant in any disability type compared to High HRQoL at the baseline. This is expected because the distribution of the disability types of Moderate-Deteriorating HRQoL was very similar to the High HRQoL at the baseline. In addition, our results provide evidence that work-limiting disabilities are associated with Group 2 and Group 4 HRQoL. The result indicates that the relative risk ratios of falling in Moderate-Improving HRQoL and Low HRQoL are significantly higher among individuals having work-limiting disabilities at the baseline than their counterparts.

This study brings a unique perspective by using preference-based HRQoL to explore its trajectory patterns and associations with clinical, socioeconomic, and demographic factors in Australia. Prior research has investigated trajectories in other well-being dimensions for people with disabilities. Australian studies identified three mental health and four subjective well-being (life satisfaction) trajectories [7, 8]. Similarly, a study from Germany observed that disability influences life satisfaction trajectories differently depending on the age of onset [10]. Our findings align with this, as Group 3 (Moderate-Deteriorating) primarily consists of older, middle-class individuals outside the workforce. Moreover, a study conducted in the UK corroborates our findings by demonstrating the adverse impact of disability onset on employment outcomes [36]. To potentially mitigate these challenges, research from the US suggests that physical activity and self-efficacy could enhance quality-of-life outcomes for elderly adults [37].

Our study builds upon the concept of the “disability paradox” explored in a Dutch study [6], which used subjective well-being measures. They compared non-declining trajectories with the paradox's existence. They found the paradox held for some subjective well-being components (depressed affect and life satisfaction) but not others (positive affect). While their approach is similar, we didn't assume a non-declining pattern as evidence for the paradox. Using preference-based HRQoL, we identified the non-declining group as having High HRQoL (Group 1). This group also has a lower prevalence of disability and higher socio-economic status. Therefore, we argue that the “disability paradox” doesn't hold in our study because we can explain the observed patterns.

### Policy implications

Our results show that, High HRQoL trajectory group, fared better outcomes than other trajectory groups. A question arises whether this group genuinely fared better than other disability groups. Since our analysis showed that High HRQoL trajectory has a lower proportion of disability distribution of all types (Fig. 3), it is safe to assume that the High HRQoL group genuinely fare better than other trajectory groups and ‘disability paradox’ actually is not a paradox. Some PwD just fare better than others. This has significant policy implications for resource allocation for health interventions. NDIS and DSP could improve allocative efficiency if group trajectories are considered.

### Strengths and limitations

The onset and nature of a disability impact a person’s overall HRQoL. However, prior research has focused on the association between HRQoL and having disability [37–39]. Other studies have examined how changes in a particular aspect of HRQoL (e.g., mental health) correlate with disability acquisition [40]. Therefore, existing studies cannot identify HRQoL trajectories of people with different types of disability. The main contribution of our study to the existing literature is showing HRQoL pathways and changes in HRQoL over time in people living with disabilities through group-based trajectory modelling. HRQoL was measured using a preference-based score (SF-6D), an additional strength of our study. Another major strength of the study is that we have studied 17 distinct forms of disability and categorize them into four broad groups to better understand how they each influence HRQoL pathways instead of focusing on disability in general. Other strengths of our study include using longitudinal data and a large sample size to precisely estimate the trajectories in HRQoL after disability onset. Our study has some limitations that need to be discussed. First, self-reported bias may arise since data on disability were collected through a self-completion questionnaire. Second, we did not consider the severity of the disability to explore its effect on HRQoL. Third, we cannot incorporate some important covariates that influence HRQoL. For example, we did not include chronic disease as a potential confounder in the model due to unavailability in all waves considered for the present study.

## Conclusion

The present study aims to identify the distinct trajectories of HRQoL for people living with disabilities and identify the relationship between different disabilities and poorer quality of life. Using group-based trajectory modelling, we found evidence that four distinct trajectories (Low, Moderate-Deteriorating, Moderate-Improving, and High) of HRQoL exist for Australian adults with a disability. We also found evidence that the relative risk of poorer quality of life (Lowest HRQoL group) is higher for people living with sensory, physical, psychosocial, and other disabilities. In addition, our findings revealed that multiple disabilities and work-limiting disability is associated with poorer quality of life. These findings reinforce the need for government policy interventions based on disability trajectories. Our findings suggest that resource allocation through NDIS and DSP for health interventions could improve allocative efficiency if group trajectories in quality of life are considered.

### Supplementary Information

Below is the link to the electronic supplementary material.

Supplementary file1 (DOCX 32 kb)

## Data Availability

Melbourne Institute of Applied Economic and Social Research (MIAESR) is the data custodian of the HILDA survey. Access to this data is restricted, and it is not publicly available. The data is available from the National Centre for Longitudinal Data (NCLD) of Department of Social Services (DSS) for researchers of approved organisations who meet the criteria for access to confidential data. Those interested in accessing this data should contact the NCLD (ncldresearch@dss.gov.au) or the Australian Data Archive (ADA, ada@anu.edu.au).
