# Supplementary material for: An investigation of the longitudinal trajectory patterns of health-related quality of life among Australians with disabilities: explaining disability types and properties
Source: Qual Life Res. 2024 Jun 10;33(8):2207–17. doi: 10.1007/s11136-024-03683-3 (PMC11286656; doi:10.1007/s11136-024-03683-3)
Supplement: Supplementary file 1 — Supplementary file1 (DOCX 32 kb) [file 11136_2024_3683_MOESM1_ESM.docx]

Appendix A

| Sl | Disability Broad Categories | HILDA Variables included | Variable definition |
| --- | --- | --- | --- |
| 1 | Sensory disability | _hespnc | Sight problems not corrected by glasses / lenses |
|  |  | _ hespch | Speech problems |
|  |  | _hehear | Hearing problems |
| 2 | Physical disability | _ hedisf | Any disfigurement or deformity |
|  |  | _ heluaf | Limited use of arms or fingers |
|  |  | _ hedgt | Difficulty gripping things |
|  |  | _ helufl | Limited use of feet or legs |
|  |  | _ hecrp | Chronic or recurring pain |
|  |  | _ hecrpa | Any condition that restricts physical activity or physical work |
| 3 | Psychosocial disability | _ hemirh | Any mental illness which requires help or supervision |
|  |  | _henec | A nervous or emotional condition which requires treatment |
| 4 | Other disability or long term conditions | _ heslu | Difficulty learning or understanding things |
|  |  | _ hesbdb | Shortness of breath or difficulty breathing |
|  |  | _ hebflc | Blackouts, fits or loss of consciousness |
|  |  | _hehibd | Long term effects as a result of a head injury, stroke or other brain damage |
|  |  | _ hemed | A long-term condition or ailment which is still restrictive even though it is being treated or medication being taken for it |
|  |  | _ heoth | Any other long-term condition such as arthritis, asthma, heart disease, Alzheimer's disease, dementia, etc |

Table A1: Definition of disability categories

Table A2: Six trajectory types modelling results with specific order (2 2 2 2 2 3 2)

|  |  |  | Standard | T for H0: |  |
| --- | --- | --- | --- | --- | --- |
| Group | Parameter | Estimate | Error | Parameter=0 | Prob > \| T\| |
|  |  |  |  |  |  |
| 1 | Intercept | 0.51 | 0.01 | 87.40 | 0.00 |
|  | Linear | -0.01 | 0.00 | -5.17 | 0.00 |
|  | Quadratic | 0.00 | 0.00 | 3.87 | 0.00 |
|  |  |  |  |  |  |
| 2 | Intercept | 0.60 | 0.00 | 185.16 | 0.00 |
|  | Linear | -0.01 | 0.00 | -4.80 | 0.00 |
|  | Quadratic | 0.00 | 0.00 | 2.71 | 0.01 |
|  |  |  |  |  |  |
| 3 | Intercept | 0.63 | 0.01 | 109.42 | 0.00 |
|  | Linear | 0.01 | 0.00 | 4.03 | 0.00 |
|  | Quadratic | -0.00 | 0.00 | -2.10 | 0.04 |
|  |  |  |  |  |  |
| 4 | Intercept | 0.74 | 0.01 | 144.84 | 0.00 |
|  | Linear | 0.01 | 0.00 | 5.05 | 0.00 |
|  | Quadratic | -0.00 | 0.00 | -6.47 | 0.00 |
|  |  |  |  |  |  |
| 5 | Intercept | 0.77 | 0.01 | 80.87 | 0.00 |
|  | Linear | -0.01 | 0.01 | -0.75 | 0.45 |
|  | Quadratic | -0.00 | 0.00 | -2.50 | 0.01 |
|  | Cubic | 0.00 | 0.00 | 2.96 | 0.00 |
|  |  |  |  |  |  |
| 6 | Intercept | 0.82 | 0.01 | 176.87 | 0.00 |
|  | Linear | 0.01 | 0.00 | 4.56 | 0.00 |
|  | Quadratic | -0.00 | 0.00 | -4.71 | 0.00 |
|  |  |  |  |  |  |
|  | Sigma | 0.08 | 0.008 | 251.81 | 0.00 |
|  |  |  |  |  |  |
| Group | membership |  |  |  |  |
| 1 | (%) | 7.82 | 0.56 | 14.05 | 0.00 |
| 2 | (%) | 28.71 | 1.01 | 28.56 | 0.00 |
| 3 | (%) | 18.88 | 1.32 | 14.36 | 0.00 |
| 4 | (%) | 19.70 | 0.84 | 23.49 | 0.00 |
| 5 | (%) | 11.64 | 1.01 | 11.70 | 0.00 |
| 6 | (%) | 13.26 | 0.78 | 16.97 | 0.00 |

Sensitivity analysis: Table A3: Factors associated with trajectory group membership in the multinomial logistic regression (Pooled).

|  | Group 1: High (Ref) | Group 2: Moderate-Improving | | | | Group 3: Moderate-Deteriorating | | |  | Group 4: Low | | |
| --- | --- | --- | --- | --- | --- | --- | --- | --- | --- | --- | --- | --- |
|  | Variables | RRR |  | 95% CI |  | RRR |  | 95% CI |  | RRR |  | 95% CI |
| Demographic | Age (Ref: 15-24 years) |  |  |  |  |  |  |  |  |  |  |  |
|  | 25-44 years | 1.78 | *** | (1.51 - 2.10) |  | 1.06 |  | (0.88 - 1.27) |  | 3.53 | *** | (2.89 - 4.33) |
|  | 45-54 years | 1.77 | *** | (1.49 - 2.10) |  | 0.93 |  | (0.77 - 1.13) |  | 3.09 | *** | (2.51 - 3.81) |
|  | 65+ years | 1.32 | ** | (1.11 - 1.57) |  | 1.28 | ** | (1.07 - 1.54) |  | 2.51 | *** | (2.05 - 3.09) |
|  | Gender (Ref: Male) |  |  |  |  |  |  |  |  |  |  |  |
|  | Female | 1.33 | *** | (1.24 - 1.42) |  | 1.17 | *** | (1.08 - 1.26) |  | 1.55 | *** | (1.46 - 1.66) |
| Clinical | Disability types |  |  |  |  |  |  |  |  |  |  |  |
|  | Sensory (Ref: No) |  |  |  |  |  |  |  |  |  |  |  |
|  | Yes | 0.86 | ** | (0.78 - 0.96) |  | 0.89 | * | (0.79 - 1.00) |  | 0.93 |  | (0.85 - 1.02) |
|  | Physical (Ref: No) |  |  |  |  |  |  |  |  |  |  |  |
|  | Yes | 1.40 | *** | (1.26 - 1.55) |  | 1.24 | *** | (1.09 - 1.40) |  | 2.67 | *** | (2.41 - 2.96) |
|  | Psychosocial (Ref: No) |  |  |  |  |  |  |  |  |  |  |  |
|  | Yes | 2.17 | *** | (1.90 - 2.47) |  | 1.84 | *** | (1.58 - 2.15) |  | 4.81 | *** | (4.26 - 5.42) |
|  | Other (ref: No) |  |  |  |  |  |  |  |  |  |  |  |
|  | Yes | 1.03 |  | (0.94 - 1.13) |  | 1.27 | *** | (1.14 - 1.41) |  | 1.64 | *** | (1.49 - 1.81) |
|  | Disability status (Ref: Single disability) |  |  |  |  |  |  |  |  |  |  |  |
|  | Multiple disability | 1.45 | *** | (1.27 - 1.66) |  | 1.42 | *** | (1.21 - 1.66) |  | 1.40 | *** | (1.23 - 1.59) |
|  | Work limiting disability (Ref: No) |  |  |  |  |  |  |  |  |  |  |  |
|  | Yes | 1.94 | *** | (1.78 - 2.12) |  | 1.60 | *** | (1.45 - 1.78) |  | 3.76 | *** | (3.46 - 4.10) |
| Socioeconomic | Income quintile (Ref: Poorest) |  |  |  |  |  |  |  |  |  |  |  |
|  | Poor | 0.85 | ** | (0.76 - 0.95) |  | 0.95 |  | (0.84 - 1.07) |  | 0.78 | *** | (0.70 - 0.86) |
|  | Middle | 0.66 | *** | (0.59 - 0.73) |  | 0.78 | *** | (0.69 - 0.89) |  | 0.51 | *** | (0.46 - 0.56) |
|  | Rich | 0.57 | *** | (0.51 - 0.64) |  | 0.71 | *** | (0.63 - 0.81) |  | 0.43 | *** | (0.39 - 0.48) |
|  | Richest | 0.47 | *** | (0.42 - 0.53) |  | 0.54 | *** | (0.47 - 0.61) |  | 0.30 | *** | (0.26 - 0.33) |
|  | Education (Ref: Year 12 or below) |  |  |  |  |  |  |  |  |  |  |  |
|  | Certificate/diploma | 1.02 |  | (0.94 - 1.10) |  | 1.01 |  | (0.93 - 1.10) |  | 0.87 | *** | (0.81 - 0.93) |
|  | Bachelor or higher | 1.05 |  | (0.96 - 1.15) |  | 0.87 | ** | (0.78 - 0.97) |  | 0.60 | *** | (0.54 - 0.65) |
|  | Labour force status (Ref: Employed) |  |  |  |  |  |  |  |  |  |  |  |
|  | Unemployed | 1.47 | *** | (1.21 - 1.78) |  | 1.43 | ** | (1.13 - 1.80) |  | 1.70 | *** | (1.37 - 2.11) |
|  | Not in the labour force | 1.32 | *** | (1.21 - 1.45) |  | 1.37 | *** | (1.24 - 1.52) |  | 2.18 | *** | (2.00 - 2.38) |
|  | Constant | 0.22 | *** | (0.18 - 0.27) |  | 0.19 | *** | (0.16 - 0.23) |  | 0.05 | *** | (0.04 - 0.07) |

RRR – Relative Risk Ratio, CI – Confidence Interval, *<0.05, **<0.01, ***<0.001

Sensitivity analysis: Table A4: Factors associated with trajectory group membership in the multinomial logistic regression (Wave 11 & age 15-64 years).

|  | Group 1: High (Ref) | Group 2: Moderate-Improving | | | | Group 3: Moderate-Deteriorating | | |  | Group 4: Low | | |
| --- | --- | --- | --- | --- | --- | --- | --- | --- | --- | --- | --- | --- |
|  | Variables | RRR |  | 95% CI |  | RRR |  | 95% CI |  | RRR |  | 95% CI |
| Demographic | Age (Ref: 15-24 years) |  |  |  |  |  |  |  |  |  |  |  |
|  | 25-44 years | 1.97 | *** | (1.33 - 2.91) |  | 1.11 |  | (0.73 - 1.69) |  | 3.64 | *** | (2.41 - 5.89) |
|  | 45-54 years | 1.60 | * | (1.06 - 2.41) |  | 0.96 |  | (0.61 - 1.52) |  | 3.02 | *** | (1.98 - 4.64) |
|  |  |  |  |  |  |  |  |  |  |  |  |  |
|  | Gender (Ref: Male) |  |  |  |  |  |  |  |  |  |  |  |
|  | Female | 1.30 | * | (1.03 - 1.63) |  | 1.10 |  | (0.83 - 1.48) |  | 1.50 | *** | (1.19 - 1.88) |
| Clinical | Disability types |  |  |  |  |  |  |  |  |  |  |  |
|  | Sensory (Ref: No) |  |  |  |  |  |  |  |  |  |  |  |
|  | Yes | 0.96 |  | (0.56 - 1.66) |  | 0.58 |  | (0.28 - 1.17) |  | 1.30 |  | (0.78 - 2.15) |
|  | Physical (Ref: No) |  |  |  |  |  |  |  |  |  |  |  |
|  | Yes | 1.68 |  | (0.97 - 2.90) |  | 0.67 |  | (0.32 - 1.43) |  | 3.53 | *** | (2.09 - 5.98) |
|  | Psychosocial (Ref: No) |  |  |  |  |  |  |  |  |  |  |  |
|  | Yes | 2.39 | ** | (1.36 - 4.20) |  | 1.04 |  | (0.47 - 2.32) |  | 5.66 | *** | (3.30 - 9.69) |
|  | Other (ref: No) |  |  |  |  |  |  |  |  |  |  |  |
|  | Yes | 1.31 |  | (0.76 - 2.26) |  | 0.81 |  | (0.38 - 1.72) |  | 2.21 | ** | (1.30 - 3.76) |
|  | Disability status (Ref: Single disability) |  |  |  |  |  |  |  |  |  |  |  |
|  | Multiple disability | 1.39 |  | (0.72 - 2.65) |  | 2.34 | * | (0.99 – 5.52) |  | 0.88 |  | (0.47 - 1.65) |
|  | Work limiting disability (Ref: No) |  |  |  |  |  |  |  |  |  |  |  |
|  | Yes | 1.96 | *** | (1.52 - 2.54) |  | 1.09 |  | (0.79 - 1.50) |  | 3.75 | *** | (2.83 - 4.98) |
| Socioeconomic | Income quintile (Ref: Poorest) |  |  |  |  |  |  |  |  |  |  |  |
|  | Poor | 0.93 |  | (0.61 - 1.41) |  | 1.03 |  | (0.59 - 1.80) |  | 0.78 |  | (0.53 - 1.15) |
|  | Middle | 0.78 |  | (0.52 - 1.13) |  | 1.02 |  | (0.62 - 1.68) |  | 0.59 | ** | (0.41 - 0.84) |
|  | Rich | 0.76 |  | (0.52 - 1.13) |  | 1.26 |  | (0.77- 2.07) |  | 0.49 | *** | (0.34 - 0.71) |
|  | Richest | 0.78 |  | (0.52 - 1.16) |  | 0.99 |  | (0.58 – 1.67) |  | 0.41 | *** | (0.28 - 0.62) |
|  | Education (Ref: Year 12 or below) |  |  |  |  |  |  |  |  |  |  |  |
|  | Certificate/diploma | 1.03 |  | (0.78 - 1.35) |  | 0.91 |  | (0.64 - 1.29) |  | 1.02 |  | (0.79 - 1.31) |
|  | Bachelor or higher | 1.03 |  | (0.75 - 1.41) |  | 0.70 |  | (0.45 - 1.07) |  | 0.69 | * | (0.50 - 0.96) |
|  | Labour force status (Ref: Employed) |  |  |  |  |  |  |  |  |  |  |  |
|  | Unemployed | 1.71 |  | (0.91 - 3.21) |  | 1.99 |  | (0.97 - 4.08) |  | 2.61 | ** | (1.42 - 4.83) |
|  | Not in the labour force | 1.67 | *** | (1.26 - 2.21) |  | 1.38 |  | (0.96 - 1.98) |  | 2.37 | *** | (1.82 - 3.11) |
|  | Constant | 0.10 | *** | (0.05 - 0.20) |  | 0.23 | *** | (0.09 - 0.57) |  | 0.02 | *** | (0.01 - 0.05) |

RRR – Relative Risk Ratio, CI – Confidence Interval, *<0.05, **<0.01, ***<0.001

Sensitivity analysis: Table A5: Factors associated with trajectory group membership in the multinomial logistic regression (Wave 11 & age 65+ years).

|  | Group 1: High (Ref) | Group 2: Moderate-Improving | | | | Group 3: Moderate-Deteriorating | | |  | Group 4: Low | | |
| --- | --- | --- | --- | --- | --- | --- | --- | --- | --- | --- | --- | --- |
|  | Variables | RRR |  | 95% CI |  | RRR |  | 95% CI |  | RRR |  | 95% CI |
| Demographic | Age (Ref: 65-74 years) |  |  |  |  |  |  |  |  |  |  |  |
|  | 75-84 years | 1.90 | ** | (1.22 - 2.96) |  | 2.31 | *** | (1.49 – 3.56) |  | 2.34 | *** | (1.58 - 3.49) |
|  | 85+ years | 2.23 | * | (1.01 - 4.93) |  | 2.17 | * | (0.99 – 4.75) |  | 2.18 | * | (1.03 - 4.59) |
|  |  |  |  |  |  |  |  |  |  |  |  |  |
|  | Gender (Ref: Male) |  |  |  |  |  |  |  |  |  |  |  |
|  | Female | 1.06 |  | (0.70 - 1.59) |  | 1.30 |  | (0.85 - 1.98) |  | 1.54 | * | (1.07 - 2.20) |
| Clinical | Disability types |  |  |  |  |  |  |  |  |  |  |  |
|  | Sensory (Ref: No) |  |  |  |  |  |  |  |  |  |  |  |
|  | Yes | 1.42 |  | (0.69 - 2.92) |  | 1.18 |  | (0.53 - 2.63) |  | 1.67 |  | (0.88 - 3.17) |
|  | Physical (Ref: No) |  |  |  |  |  |  |  |  |  |  |  |
|  | Yes | 1.96 |  | (0.90 - 4.30) |  | 1.02 |  | (0.45 - 2.32) |  | 3.34 | *** | (1.68 - 6.64) |
|  | Psychosocial (Ref: No) |  |  |  |  |  |  |  |  |  |  |  |
|  | Yes | 7.82 | ** | (1.83-33.45) |  | 4.73 | * | (1.03 - 21.77) |  | 13.23 | *** | (3.36-52.05) |
|  | Other (ref: No) |  |  |  |  |  |  |  |  |  |  |  |
|  | Yes | 2.21 |  | (0.98 - 5.02) |  | 1.06 |  | (0.46 - 2.43) |  | 3.00 | ** | (1.47 - 6.11) |
|  | Disability status (Ref: Single disability) |  |  |  |  |  |  |  |  |  |  |  |
|  | Multiple disability | 0.57 |  | (0.22 - 1.46) |  | 0.74 |  | (0.28 - 1.97) |  | 0.72 |  | (0.32 - 1.62) |
|  | Work limiting disability (Ref: No) |  |  |  |  |  |  |  |  |  |  |  |
|  | Yes | 2.41 | *** | (1.53 - 3.79) |  | 1.67 | * | (1.11 - 2.51) |  | 5.77 | *** | (3.81 - 8.75) |
| Socioeconomic | Income quintile (Ref: Poorest) |  |  |  |  |  |  |  |  |  |  |  |
|  | Poor | 0.93 |  | (0.54 - 1.60) |  | 0.83 |  | (0.48 - 1.46) |  | 0.84 |  | (0.52 - 1.36) |
|  | Middle | 0.50 | ** | (0.29 - 0.85) |  | 0.48 | ** | (0.28 - 0.83) |  | 0.38 | *** | (0.24 - 0.60) |
|  | Rich | 0.50 | * | (0.26 - 0.99) |  | 0.60 |  | (0.31- 1.17) |  | 0.58 |  | (0.32 - 1.03) |
|  | Richest | 0.36 | ** | (0.17 - 0.76) |  | 0.70 |  | (0.37 - 1.33) |  | 0.30 | *** | (0.15 - 0.58) |
|  | Education (Ref: Year 12 or below) |  |  |  |  |  |  |  |  |  |  |  |
|  | Certificate/diploma | 1.03 |  | (0.65 - 1.63) |  | 1.31 |  | (0.83 - 2.06) |  | 0.78 |  | (0.52 - 1.17) |
|  | Bachelor or higher | 0.82 |  | (0.46 - 1.47) |  | 0.81 |  | (0.45 - 1.46) |  | 0.56 | * | (0.34 - 0.93) |
|  | Labour force status (Ref: Employed) |  |  |  |  |  |  |  |  |  |  |  |
|  | Unemployed | 0.00 | *** | (0.00 - 0.00) |  | 0.00 | *** | (0.00 - 0.00) |  | 3.18 |  | (0.60-16.77) |
|  | Not in the labour force | 1.09 |  | (0.57 - 2.10) |  | 1.19 |  | (0.62 - 2.27) |  | 1.22 |  | (0.67 - 2.20) |
|  | Constant | 0.17 | *** | (0.06 - 0.49) |  | 0.33 | * | (0.11 - 0.96) |  | 0.08 | *** | (0.03 - 0.22) |

RRR – Relative Risk Ratio, CI – Confidence Interval, *<0.05, **<0.01, ***<0.001

Appendix B

Group Based trajectory modelling:

Let, $H_{i}=\left\{ h_{i1},h_{i2},\ldots h_{it}\ldots,h_{iT} \right\}$ be a sequence of longitudinal outcome for individual $i$ over $T$ periods. Then GBTM assumes that the population distribution of trajectories of some outcome $H_{it}$ arises from a finite mixture of unknown order $j$ such that:

$P\left( H_{i}|t \right)= \sum_{j} \theta_{j}P_{j}(H_{i}|t,\beta^{j})$ (1)

Where, $P\left( H_{i}|t \right)$ is the distribution of outcome trajectories for $H_{i}$, $P_{j}(H_{i}|t,\beta^{j})$ is the probability of $H_{i}$ given membership of group $j$ and $\theta_{j}$ is the probability of group $j$. Conditional independence of realization $H_{i}$ is assumed, so that, $P_{j}\left( H_{i} | t,\beta^{j} \right)= \prod_{t} P_{j}(H_{i}|\beta^{j})$. Group membership probabilities are estimated by a generalized logistic function with $\lambda_{1}=0$:

$\theta_{j}= \frac{e^{\lambda_{j}}}{\sum_{j} e^{\lambda_{j}}}$ (2)

The form of $P_{j}(H_{i}|t,\beta^{j})$ can be poisson distribution if the outcome is a count variable, logit distribution for binary data and censored normal for scaled data. Since our outcome variable is scaled data, we used the censored normal distribution in our specification. Thus, assuming cubic polynomial order (the order defines the shape of the trajectories and could be higher or lower order), the likelihood of observing a trajectory of group $j$ :

$h_{it}^{*j}=\beta_{0}^{j}+{\beta_{1}^{j}{Time}_{it}+ \beta_{1}^{j}{Time}_{it}^{2}+ \beta}_{1}^{j}{Time}_{it}^{3}+ \varepsilon_{it}$ (3)

Estimation of trajectory groups:

The number of trajectory groups was determined using the following procedure:

Step 1: We began with a cubic functional order and evaluated the Bayes factor of each successive model until the test statistic threshold fell below specified threshold.

Step 2: In addition, we examined whether the following statistic fell below the cutoff: entropy<0.8, group size<0.05, APP<0.7 and OCC<5.

Step 3: We visualize the trajectories and see whether adding an extra group provides any meaningful groups.

Step 4. Evaluating all criteria, we select the group that is most plausible.

Step 5: Once we selected the group, we check the p values of the orders to determine the shape. If a higher order is significant in a particular group, we select that order. If the current order is not significant, we lower the order until it is significant.

Step 6. Once trajectories were identified, we again checked whether the groups were providing any extra information or whether trajectories could be merged.

Step 7: We performed a descriptive analysis of the identified groups.

Step 8: We performed a multinomial logistic regression to profile individual characteristics of the identified groups.
